# Mining-Induced Time-Series Deformation Investigation Based on SBAS-InSAR Technique: A Case Study of Drilling Water Solution Rock Salt Mine

**DOI:** 10.3390/s19245511

**Published:** 2019-12-13

**Authors:** Xiangbin Liu, Xuemin Xing, Debao Wen, Lifu Chen, Zhihui Yuan, Bin Liu, Jianbo Tan

**Affiliations:** 1 Laboratory of Radar Remote Sensing Applications, Changsha University of Science and Technology, Changsha 410014, China; liuxb0219@foxmail.com (X.L.); lifu_chen@139.com (L.C.); yuanzhihui@csust.edu.cn (Z.Y.); binliu@csust.edu.cn (B.L.); tanjianbo@imde.ac.cn (J.T.); 2 School of Traffic and Transportation Engineering, Changsha University of Science and Technology, Changsha 410014, China; 3 School of Geographical Sciences, Guangzhou University, Guangzhou 510006, China; wdbwhigg@gzhu.edu.cn; 4 School of Electrical and Information Engineering, Changsha University of Science and Technology, Changsha 410014, China

**Keywords:** SBAS-InSAR, deformation, rock salt mine, drilling solution mining, time series

## Abstract

Compared to traditional coal mines, the mining-induced dynamic deformation of drilling solution mining activities may result in even more serious damage to surface buildings and infrastructures due to the different exploitation mode. Therefore, long-term dynamic monitoring and analysis of rock salt mines is extremely important for preventing potential geological damages. In this work, the small baseline subset Interferometric Synthetic Aperture Radar (SBAS-InSAR) technique with Sentinel−1A imagery is utilized to monitor the ground surface deformation of a rock salt mining area. The time-series analysis is carried out to obtain the spatial–temporal characteristics of land subsidence caused by drilling solution mining activities. A typical rock salt mine in Changde, China is selected as the test site. Twenty-four scenes of Sentinel−1A image data acquired from June 2015 to January 2017 are used to obtain the time-series subsidence of the test mine. The temporal–spatial evolution of the derived settlement funnels is revealed. The time-series deformation on typical feature points has been analyzed. Experimental results show that the obtained drilling solution mining-induced subsidence has a spatial characteristic of multiplied peaks along the transversal direction. Temporally, the large-scale surface settlement for the rock salt mine area begins to appear in September 2016, with a time lag of 8 months, and shows an obvious seasonal fluctuation. The maximum cumulative subsidence is detected up to 199 mm. These subsiding characteristics are consistent with the connected groove mining method used in drilling water solution mines. To evaluate the reliability of the results, the SBAS-derived results are compared with the field-leveling measurements. The estimated root mean square error (RMSE) of ±11 mm indicates a high consistency.

## 1. Introduction

**Figure 1:**
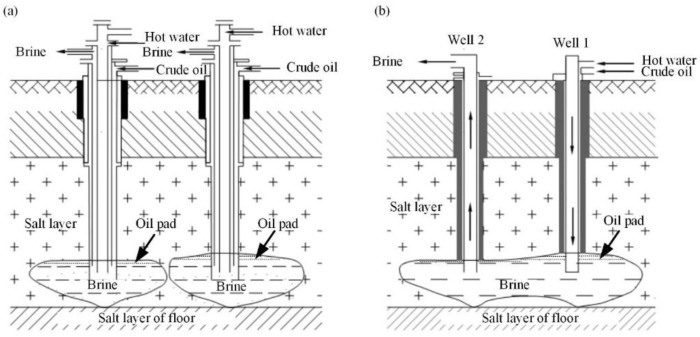
(**a**) Single well based on an oil pad before the process of dissolution connection, (**b**) The final connected groove based on an oil pad after the process of dissolution connection process (data from [2]).

The reserves of mirabilite deposits of China had been proved to be accumulated up to 1117.20 billion tons until the end of 2017 [1]. An omnidirectional advanced drilling solution mining is the dominate exploitation method for most mirabilite mines [2]. The connected groove mining method based on an oil pad is applied for most of the drilling solution mining activities. Figure 1 shows the schematic diagram of the connected groove mining method with two salt wells. It can be seen that each salt well is built on an oil pad with a dissolution cavity in the mining layer. The single well based on an oil pad is used to build grooves in the early stages of drilling solution mining (see Figure 1a), which can promote the side dissolution, control the upper dissolution, and speed up the connection of well groups. As the process of dissolution, the cavities derived by adjacent salt wells can be connected and merged in the mining layer (see Figure 1b). After the process of connection dissolution between different cavities, the fresh water at 40 °C is injected through one of the wells (demonstrated as well 1 in Figure 1). Subsequently, the mirabilite layer can be dissolved, and under the water injection residual pressure, the generated brine can be cramped out from the other well (demonstrated as well 2 in Figure 1) [2]. Due to the long time of the dissolution process and the certain supporting effect of the high-pressure injected fresh water to the roof, time lag and suddenness are the obvious characteristics of the ground deformation related to drilling solution mining activities. Compared to tunnel mining with the unidirectional propulsion of conventional coal mines, the depth of drilling solution mining is generally much deeper, and the thickness of the rock salt layer is even thicker. With a serious influence imposed by the water on the mechanical properties for the salt roof, the subsidence related to drilling solution mining will be even more severe and destructive [3]. Due to the omnidirectionality and uncertainty of drilling solution mining, the mechanical properties of the cavity may become unreasonable, which may induce the overburden or even serious collapse on the cavity [4,5]. Once the roof of cavity reaches the bottom ground, a sinkhole will be generated at the surface [6,7], which may induce potential damage to the nearby infrastructures (i.e., houses, roads, bridges, canals) [8]. Furthermore, the sustained mining of rock salt mines can easily lead to mechanical changes to the underground rock and water system. This may even cause brine pumping and land salinizing [9], which shows serious potential for environmental pollution [10]. Therefore, the long-term spatial–temporal deformation monitoring of rock salt mines is of practical significance to the prevention of mining-induced safety problems and the assurance of mining environmental protection.

Traditional geodetic monitoring methods, such as total station/prism, photogrammetry, leveling, and Global Navigation Satellite System (GNSS), have been widely applied in mining-induced deformation monitoring. Those methods are proven to be of high accuracy. However, due to the poor spatial–temporal resolution, those methods still have deficiencies in observing the overall ground surface subsidence of the mining area [11]. In addition, expensive labor force, and frequently in situ observation are necessary for the monitoring of mining area, which will consume an enormous amount of financial resources and inevitably aggravate the potential safety problems.

Interferometric Synthetic Aperture Radar (InSAR) offers a novel earth observation approach. It can provide wide spatial coverage, high imaging resolution, and non-intrusive surveying. Differential InSAR (D-InSAR), as an extension of InSAR in terms of monitoring ground deformation, is mainly applied in ground deformation monitoring along the line of sight (LOS) of a radar satellite. The new monitoring approach is an important complement to the traditional geodetic surveying methods [12]. D-InSAR is widely applied to detect and monitor earthquake deformation [13], glacial shift [14], volcanic activity [15], and landslides [16], as well as man-made activities such as mining subsidence [17] and urban settlement caused by groundwater overdraft [18]. However, the unavoidable influences of the temporal and spatial decorrelation and atmospheric delay have brought restrictions on its application, especially on mining areas vulnerable to decorrelation. Small baseline subset InSAR (SBAS-InSAR) is an advanced InSAR technology proposed by Berardino [19], which utilizes the least squares (LS) and singular value decomposition (SVD) methods to obtain the deformation rates at the high coherence points based on the multi-scene of differential interferometric images. Although a large amount of successful cases using SBAS technology in coal mine areas have been published [20,21], the application in rock salt drilling solution mining has been rarely mentioned in previous studies.

The Sentinel−1 satellite, equipped with a C-band SAR sensor, is an Earth observation satellite launched by the European Space Agency’s Copernicus Program in 2014. Sentinel−1A SAR data have the advantages of large global coverage and a short revisit period (12 days), which can be downloaded free of charge on the website (https://scihub.copernicus.eu/) [22]. Sentinel−1A SAR data have been widely and successfully applied in the monitoring of mining-induced subsidence [23,24]. In this work, a typical rock salt mine in Changde, China was selected as the test site. In order to verify the feasibility and reliability of the SBAS technique and Sentinel−1A imagery for the deformation monitoring of rock salt mines, we use SBAS and Sentinel−1A images to perform a case study. The time-series characteristics of the subsidence sequences related to drilling solution mining activities are revealed.

## 2. Methodology

Suppose *N +* 1 SAR images covering the same area are acquired in repeat orbits at different dates $\left(T_{0},T_{1},\;\ldots ,T_{N}\right)$. Then, M interferometric pairs can be produced according to certain spatial–temporal baseline thresholds, where M satisfies the inequality (N+1)/2≤M≤N(N+1)/2. Each of these interferometric pairs is generated by the two-orbit D-InSAR processing. In the processing, all images are registered and resampled to the same image first. Then, an external digital elevation model (DEM) is used to remove the topographic phase, and consequently, phase unwrapping is carried out for each interferometric pair. The unwrapped phase at pixel (x, r) in the *i*-th (i=1, 2, …, N) interferogram can be written as [19].

(1) $$\delta \varphi_{i}=\phi_{B}\left(x,r\right)-\phi_{A}\left(x,r\right)\approx \frac{4\pi }{\lambda }\Delta d+\frac{4\pi B_{⊥}}{\lambda r\;sin\;\theta }\Delta h\left(x,r\right)+\Delta \varphi_{i,res\left(x,r\right)}$$

where *λ*, θ, and $B_{⊥}$ represent the SAR coordinate of the high coherence point, the radar wavelength, the radar incidence angle, and the perpendicular baseline of the two SAR acquisitions, respectively; $\Delta d=d\left(T_{A},x,r\right)-d\left(T_{B},x,r\right)$ is the time-series displacements along the LOS direction at date $T_{A}$ and $T_{B}$ respectively, with respect to the start time (i.e., $d\left(T_{0},x,r\right)\equiv 0$); $\Delta \varphi_{i,res\left(x,r\right)}$ is the residual phase, including the phase noise, the atmospheric delay, and the high-pass (HP) deformation component; Δh(x,r) represents the topographic error of the external DEM.

**Figure 2:**
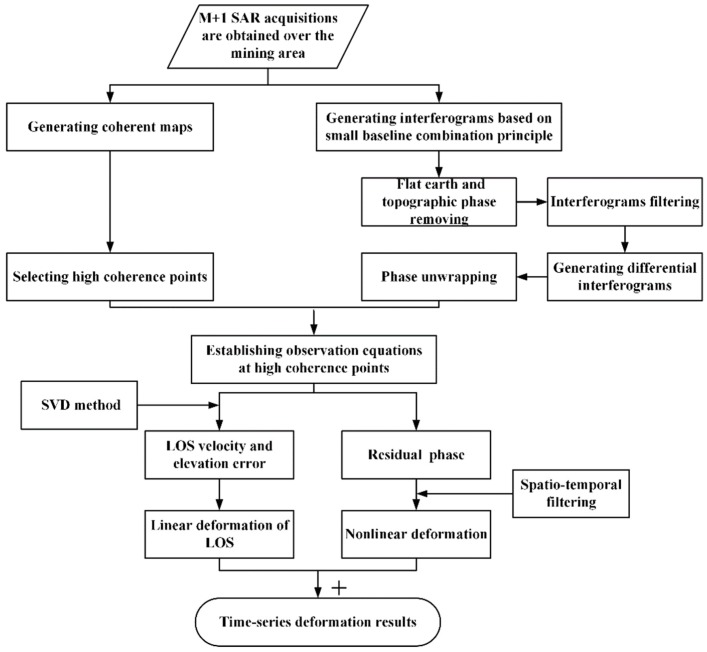
Experimental flow of small baseline subset (SBAS) algorithm.

The deformation component Δd is of the main interest. The functional relationship between Δd and the deformation parameters can be written as [25]

(2) $$\Delta d=\overset{s}{\underset{k=l+1}{\sum }}v_{k}\left(T_{k}-T_{k-1}\right)$$

where l and s define the index of the master image at time $T_{A}$ and slave image at time $T_{B}$, respectively for the *i*-th interferometric pair. $v_{k}$ defines the linear velocity for each temporal unit, which varies across different temporal units. According to Equations (1) and (2), we need to estimate *N* number of vs. as well as the unknown DEM error parameter Δh (in total N + 1 unknown parameters) in M generated functions. To solve the singular mathematical problem, the SVD algorithm and LS method are suggested here [26,27]. After the unknown parameter being estimated (v and Δh), integration over each temporal period is carried out to obtain the low-pass (LP) deformation component on all the high coherence points. Considering that the atmospheric delay phase component is a temporally random high frequency signal, it is spatially related to the low frequency signal. In contrast, the nonlinear deformation phase is a low-frequency signal both spatially and temporally [28,29]. Accordingly, in order to pick up the HP deformation component from the residual phase, a temporally high-pass filtering and a spatially low-pass filtering need to be applied. The final deformation on each coherent point will be obtained through summarizing both the LP deformation and HP deformation. The experimental flow of SBAS processing is shown in Figure 2.

In this work, the LOS deformation is converted to the vertical component in order to compare with the in situ leveling measurements (the horizontal displacement is omitted in the experiment, which will be discussed in Section 3.1), according to the following function [30]:

(3) $${Def}_{vertical}{=Def}_{LOS}/\cos\theta$$

where ${Def}_{LOS}$ represents the LOS deformation and ${Def}_{vertical}$ represents the vertical component.

## 3. Experiments

### 3.1. Study Area and Geological Background

**Figure 3:**
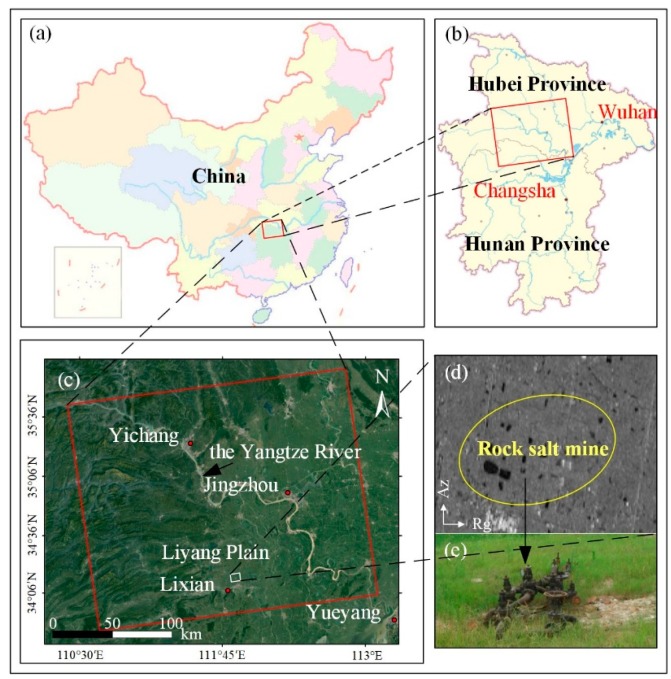
Study area overview. (**a**,**b**) Regional scale in China of the test mine. (**c**) The location of the study area. (**d**) Corresponding amplitude image of the area with the mining region of interest outlined in the white rectangle. (**e**) In situ picture of the drilling solution wellhead in (**d**).

**Figure 4:**
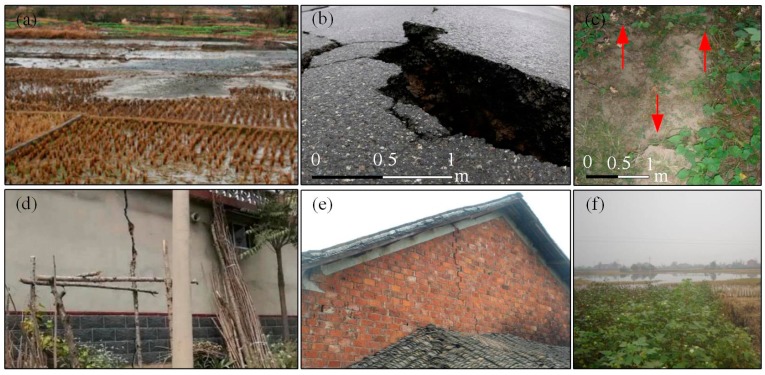
In situ pictures of ground ruptures in the rock salt mine. (**a**) The underground brine flowing into the cultivated land, inducing land salinization. (**b**,**c**) Accumulated deformation induced road surface cracks. (**d**,**e**) Cracks on resident houses (**f**) Gradually formed stagnant water area, with a settlement even deeper than 1.5 m.

In this work, a typical water-soluble rock salt mine in Changde, Hunan Province is selected as our test site. Figure 3 shows the location of the test area. Figure 3a,b shows the corresponding study areas on a map of China. Figure 3c shows the optical images of the mine area. It can be seen from Figure 3c that the rock salt mine area is located in the Liyang Plain of Hunan Province, with a total area of 5.7 km^2^. The red rectangle represents the spatial coverage of ascending Sentinel−1A images, whereas the white rectangle is the selected subset of interest in this work. Due to the location in the middle of the plain, the rock salt mine has a typical flat terrain characteristic, surrounded with dense pounds, natural water systems, and artificial channels. It is also located close to a wide area of surface rice fields. Since 2002, the long-term mining activities in this area have caused great damage to the surrounding environment and underground geological stratum (see Figure 4).

**Figure 5:**
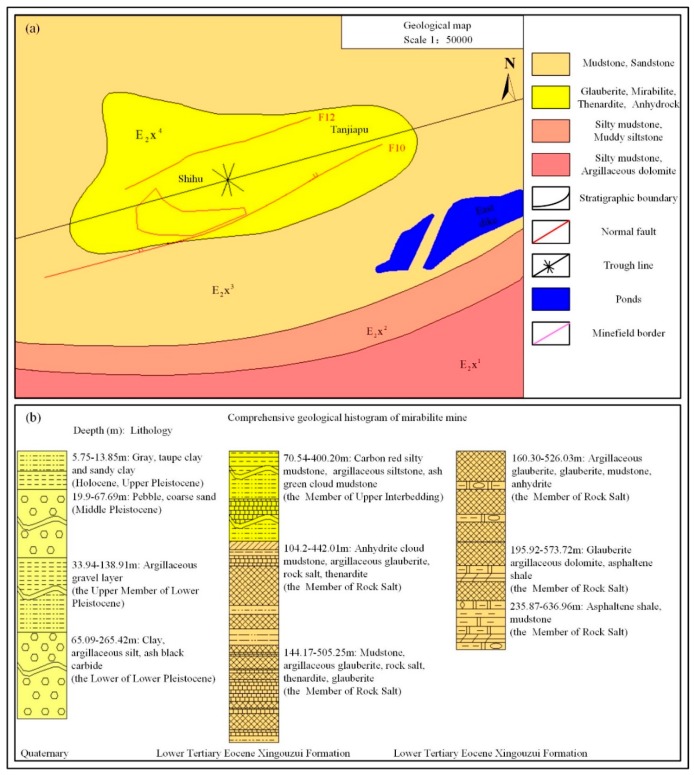
Geological map of the rock salt mine. (**a**) The plane geological distribution of the test rock salt mine ($E_{2}X$ represents the Lower Tertiary Eocene Xingouzui Formation). (**b**) Vertical distribution of the comprehensive geological formations (data from [31]).

Figure 5 illustrates the geological distribution of the test rock salt mine [31]. The strata encountered in drilling solution mining mainly includes the Quaternary System and Lower Tertiary System. The distribution of the strata from top to bottom is as follows: Holocene, Upper Pleistocene, Middle Pleistocene, and Lower Pleistocene. The total thickness of the strata is 77.95–138.55 m. The Lower Tertiary System consists of Eocene and Paleocene, with a total thickness of 562.96 m. The lithology of the Eocene Formation mainly includes mudstone, dolomite, siltstone, gypsum, glauberite, and thenardite. The extracted thenardite (Na_2_SO_4_, 62.76%–78.8%) and mirabilite of this mine are present in the salt-bearing section of the Xingouzui Formation of the Tertiary System (E_2_x^3^), with a cumulative thickness of 8.21–14.23 m. The fault structure of the mining area is mainly F10 fault, located in the south, with a stratum fault distance of 30–70 m and a 3–16 cm fracture zone. It is filled with fibrous gypsum cementation. After the south plate rises, F10 destroys the continuity of the seam in the south wing of the syncline and causes the minerals to dissolve. Therefore, F10 constitutes the natural boundary in the south of the mining area. A concealed fault F12, with a dip angle of 75°, belongs to the SEE (South East East) normal fault. The roof, floor, and interlayer of the ore bed, containing a small amount of anhydrite dolomitic mudstone, are mainly muddy dolomitic glauberite, which belongs to the weak layered rock mass. The rock, with poor stability, is easy to soften and collapse. 

In order to prevent mining accidents and natural environment pollution caused by mining-induced roof collapse, hot water combined with the connected groove mining method based on an oil pad is utilized to extract thenardite in this salt mine. During the period from June 2016 to January 2017, the groove connection was completed in the test mine. Different cavities below different wells were mutually dissolved and connected (see Figure 1). The dissolution and transport channels of minerals were formed during this period. Since then, the stage of the upper dissolution started. The shape of the cavity started to change along the upper direction, which developed along the vertical deeper direction. During this stage, the side dissolution rate was significantly reduced, whereas the upper dissolution rate became twice as fast as the side dissolution, which performed as a significant ground subsidence along the vertical direction [32]. Therefore, the horizontal displacement is omitted in our experiment.

### 3.2. SAR Acquisitions and Data Processing

A total of 24 repeat-pass ascending Sentinel−1A images of the test rock salt mine area were collected. These acquisitions covered the period from 15 June 2015 to 30 December 2016. The parameters of these images are listed in Table 1. SARScape 5.2 and ENVI 5.3 were used to generate the unwrapped small baseline interferometric pairs. The subsequent procedures, including high coherence points identification and the LP–HP deformation component estimation, were carried out through MATLAB.

**Figure 6:**
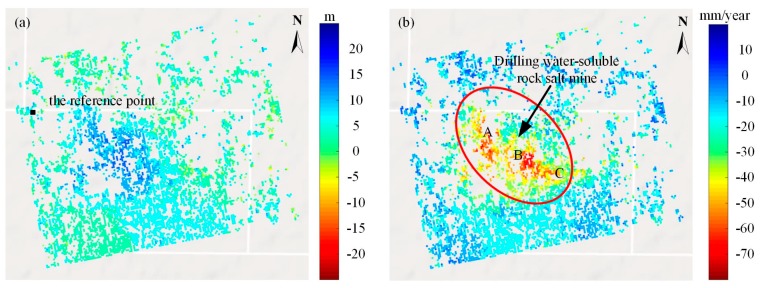
(**a**) Digital elevation models (DEM) errors (the solid black square represents the reference point for phase unwrapping). (**b**) Deformation rates.

The thresholds for the spatial–temporal baseline of the interferometric combination were empirically set to 150 m and 360 days, respectively. In the two-pass D-InSAR processing, all the rest of the images were registered and resampled to the super master image. In order to remove the topographic phase, a 1-arc-second Shuttle Radar Topography Mission Digital Elevation Model (SRTM DEM, ~30 m spacing) provided by NASA was utilized. In addition, a Gaussian filter [33] was selected to suppress the phase noise. After the flat Earth phase removal and phase filtering processing, a polynomial fitting method was used to remove the orbital error; then, a relatively stable reference point was selected (see Figure 6a) and minimum cost flow [34] method was utilized to unwrap the wrapped interferometric deformation phases. Finally, a total of 58 unwrapped differential interferograms were generated. During the processing, based on a coherence threshold of 0.45 and amplitude dispersion threshold of 0.35, a total of 5559 high coherence candidates for the test mine were selected. 

## 4. Results and Discussion

### 4.1. Overall Deformation Results

The DEM error and the linear deformation rate of the high coherence points were obtained, which are shown in Figure 6. Through our quantitative analysis and statistics, the total number of the high coherence points, with the absolute DEM error values within the range of 0 m to 10 m, account for 86%, whereas the number within the range of 10 m to 20 m only account for 14% (blue to green color, as shown in Figure 6a). It is in good agreement with the accuracy of SRTM DEM data with 30-m resolution [35]. From Figure 6b, we can see an obvious subsidence bowl in the central region where the rock salt mine is located, with the color gradually changing from blue to red inwardly. According to our analysis, the subsidence rate of most coherence points is distributed within the range of 50 mm/year to 75 mm/year, with the maximum value of up to 109 mm/year.

According to [36], the accuracy of the retrieved topographic residuals is related to the thresholds of the perpendicular baseline and the quality of the differential interferogram. The accuracy of the DEM error will degrade the accuracy of the InSAR time-series deformation. Due to this, we controlled the spatial baseline threshold and selected the interferometric pairs strictly (the threshold was set as 150 m in our experiment). The external SRTM DEM had 30 m resolution, which is relatively high. Moreover, in order to show the correlation of the DEM error and the deformation, we conducted a simulated experiment. According to the phase contribution of DEM error, $\delta \varphi =\frac{4\pi }{\lambda }\frac{B_{⊥}}{R\;sin\;\theta }\cdot \Delta h$, and the relationship between phase and the deformation velocity, $\delta \varphi =\frac{4\pi }{\lambda }v\left(T_{B}-T_{A}\right)$, the error of deformation velocity caused by a 20 m DEM error was only 5 mm/year. Consequently, compared to the large estimated subsidence (a maximum deformation velocity of 109 mm/year), the influence of DEM error on the deformation time series was ignored in our case study.

**Figure 7:**
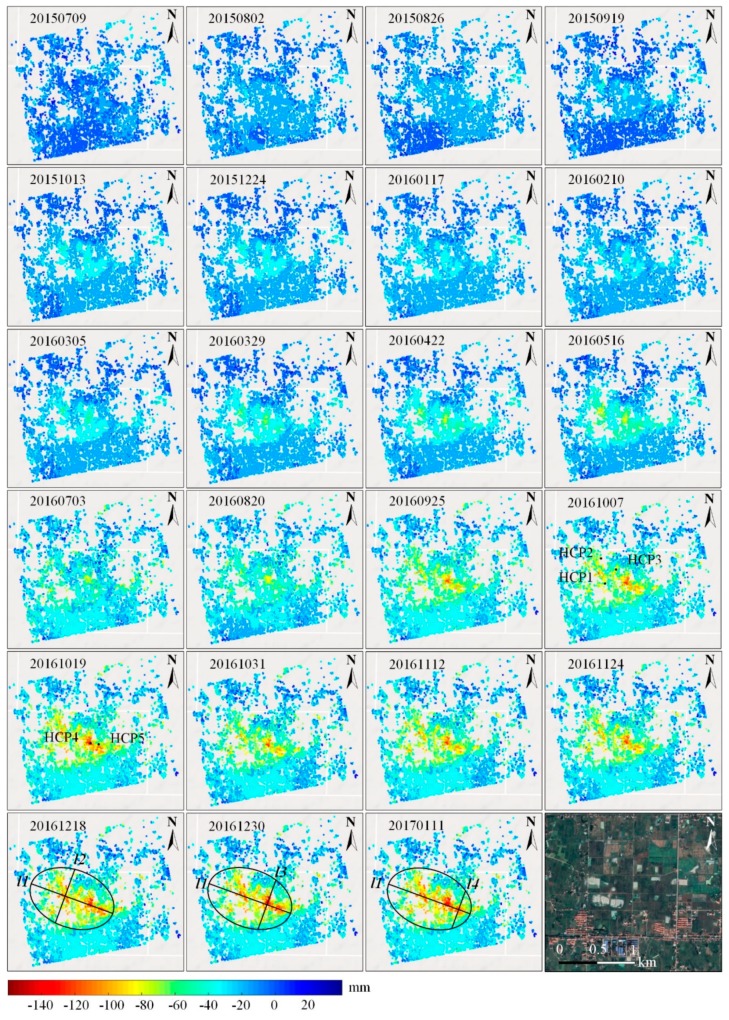
Time-series deformation (with reference to 15 June 2015).

Figure 7 shows the overall time-series deformation of the rock salt mine. From the spatial characteristics of the color distribution, we can see that the obvious subsiding points were densely distributed in the center part of the images, where the rock salt mine was located (with a dark orange to red color in the subsidence bowl). The spatial distribution characteristics of the subsiding pixels in the mining area appeared as disperse zonal distribution in the northwest part and an overall sheet-like distribution in the central and southeast part. The reason for this phenomenon is that the drilling solution mining method based on connected well groups was utilized in the middle and southeast part, which induced a dissolution connection of different cavities underground; thus, the surface subsidence performed to be multi-subsiding bowls (see A, B, and C in Figure 6). Meanwhile, in the northwest of the area, the single well drilling solution method was adopted, which resulted in disperse zonal distribution characteristics.

As the temporal color variation shows in Figure 7, a temporal characteristic of seasonal fluctuation could be found (which will be analyzed quantitively in Section 4.2). For the period from 9 July 2015 to 10 February 2016, the subsidence velocity was relatively stable (which will be mentioned as the time lag in Section 4.2). From 5 March to 3 July 2016, a slow increase of subsiding occurred, while for the period from 3 July to 19 October 2016, a rapid subsiding dominated the deformation. The subsidence bowl started to appear on 5 March 2016. Since then, an obvious large subsiding velocity began to occur in the mining area. By 11 January 2017, the maximum subsidence in the bowl was accumulated to 199 mm.

By September 2016, large-scale subsidence began to occur. Subsidence bowls A, B, and C were gradually generated by rapid mining activities through multiple wells. Since B and C were connected by well groups, the caverns were interconnected underground. As the increasing of mineral exploitation, the volume of the caverns increased gradually, leading to more serious movements on the top edge of the chamber and closer distance between different caverns. Sequentially, funnels B and C would be merged into a large subsidence bowl.

### 4.2. Discussions

As discussed in Section 4.1, the overall time-series deformational characteristics of the test rock salt mine follows spatially multi-distributed bowls and a temporal 8-month time lag, with a subsequent annual fluctuation. The reasons for the subsiding characteristics are supposed to be as follows:

(1) The process of the brine extraction was conducted by injecting solvent followed by rock salt dissolution, which takes a longer time than traditional coal mining activities; in addition, the depth of the drilling solution mining was deeper than that of common coal mines (the depth of wells in this study area was 200–500 m), which induced the lagging appearance of ground surface subsidence.
(2) The relationship between the solubility and the solvent temperature in Table 2 shows that the dissolution of mirabilite is significantly vulnerable to temperature [2,37]. The solubility under 30 °C is almost four times that of under 0–10 °C. This indicates that under the circumstance of high temperature in the warm season, the mineral dissolution was considerably rapid, inducing a larger amount of brine extraction. On the contrary, for the cold season (the period of 24 December 2015 to 25 March 2016), the low temperature in winter suppressed the dissolution rate for the mirabilite.
(3) The spatially multi-peak phenomenon was mainly due to the drilling solution mining method based on connected well groups and its comprehensively multi-direction advancing mode (which will be discussed in Section 4.2).

**Figure 8:**
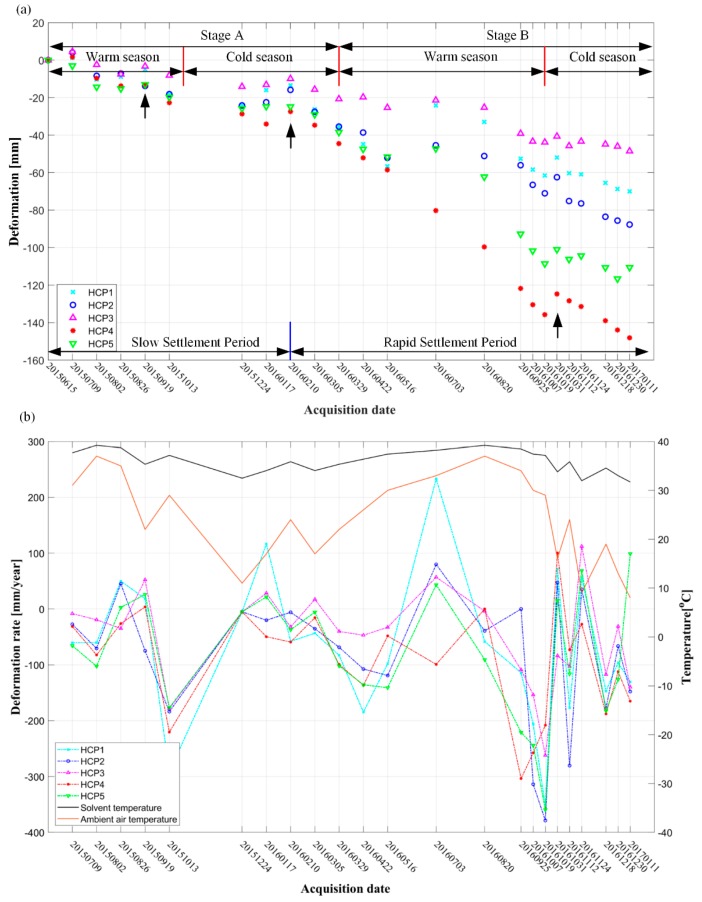
(**a**) Time-series deformation of feature points at drilling water-soluble rock salt mine (HCP1 to HCP5). (**b**) Correlation diagram of the deformation rate with the solvent temperature and the average air temperature.

In order to further reveal the characteristics of temporal deformation variation, five feature points (HCP1 to HCP5 shown in Figure 7) were selected for quantitive analysis. The extracted time-series deformation is illustrated in Figure 8a.

**Figure 9:**
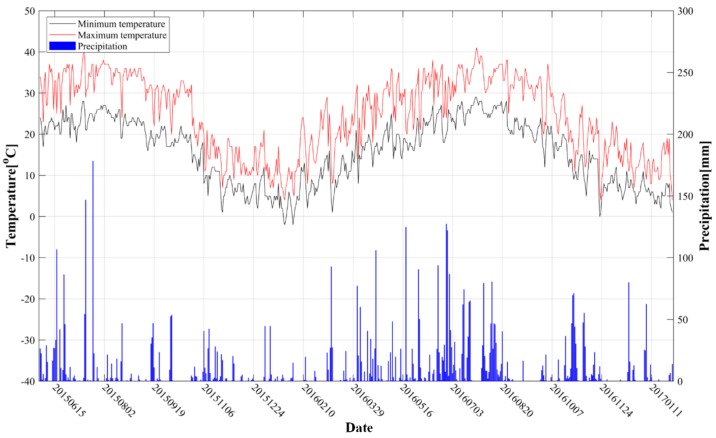
The temperature and precipitation in the study area (from 6 June 2016 to 31 January 2017).

From Figure 8a, we can see for the total period (15 June 2015 to 11 January 2017) that all the five feature points show similar temporal variations: a generally subsiding trend with an obvious seasonal fluctuation. HCP4 showed the most serious subsiding, with an accumulative subsidence of 148 mm until 11 January 2017, whereas HCP3 was relatively more stable, with the maximum deformation of 48 mm. For the cold season from 24 December 2015 to 10 February 2016 in stage A, and 19 October 2016 to 1 January 2017 in stage B, a relatively slow deformation trend occurred, with a small fluctuation of 11 mm and 12 mm, respectively. From 29 March 2016, a significant subsiding trend started. The seasonal fluctuation of the deformation in a rock salt mine was suggested to be mainly due to the dissolution rate of mirabilite and thenardite in water. The dissolution rate of thenardite was directly affected by the temperature of the solvent. In the production process of the rock salt mine, combined with the connected groove mining method based on an oil pad, hot water was suggested to be used as solvent to increase the dissolution. The hot water transported from the processing industry was pressurized by the injection pump, metered at the control station, and then directly injected into the well after distribution. According to our investigation, the temperature of the fresh water solvent injected into the well was about 40 °C. However, during the transportation from the processing industry and the injection process into the well through the injection pump, the solvent temperature was vulnerable to the air temperature. In summer, the temperature of fresh water could be well insulated. On the contrary, in winter, due to the decrease of external temperature, the temperature of fresh water was easy to decrease. Consequently, the high temperature in the warm season accelerated the dissolution of rock salt, which lead to the increase of subsidence. In contrast, the low temperature in cold seasons suppressed the process of water dissolution, inducing the slow or even uplifting trend of deformation. Three small uplifts for the five feature points can be found in the position pointed by the black arrows in Figure 8a, which showed good consistency with the measurements detected from Figure 7. The uplift phenomenon was mainly related to the aforementioned low temperature in cold seasons and the increase of rainfall (from 26 August to 19 September 2015, 19 October to 31 October 2016, and 17 January to 10 February 2016, as shown in Figure 9) [38].

In order to further prove the aforementioned hypothesis that the seasonal fluctuation of our obtained deformation was related to the temperature of solvent, we tried to obtain the temperature of the solvent during our observation period. Due to the limitation of the unavailable solvent temperature data, we used the principle of heat transfer and hydrodynamics introduced in [39] to derive the temperature of the solvent in our experiment. The temperature difference between the pipe inlet and outlet water can be written as:

(4) $$\Delta t=t_{g}-t_{o}=\frac{k_{g}L\left(t_{p}-t_{k}\right)}{GC}$$

where $t_{g}$ represent the temperature of the pipe inlet water, which was treated as a constant (40 °C in our experiment); $t_{o}$ represents the pipeline outlet water temperature, which was the unknown temperature of the solvent injected into the cavity; $k_{g}$ is the heat transfer coefficient of the pipeline, which could be indexed according to the pipe material; $t_{p}$ is the average temperature, which could be calculated according to $t_{p}=\left(t_{g}+t_{o}\right)/2$; $t_{k}$ is the ambient air temperature; L is the length of the pipeline, G is the mass flow of hot water (both L and G could be provided by the mining company); and C is the specific heat capacity of the hot water, which could be indexed from the standard industry document provided by the mining company.

Formula (4) can be transferred to

(5) $$t_{g}-t_{o}=\frac{k_{g}L\left(\left(t_{g}+t_{o}\right)/2-t_{k}\right)}{GC}$$

and then $t_{o}$ can be estimated through Equation (5).

We added the obtained solvent temperature into the correlation analysis between the deformation velocities and the external air temperature, which is shown in Figure 8b. The five feature points on the graph are the time-series settlement points mentioned above. As can be seen from the figure, the temperature of the solvent was highly related to the external air temperature, and the linear deformation velocity also showed high correlation with the solvent temperature. In warm seasons, the subsidence rates of the mining area increased with the air temperature, whereas in cold seasons, the subsidence rates showed obvious dropping with the decrease of the temperature. This result proves the aforementioned hypothesis. 

**Figure 10:**
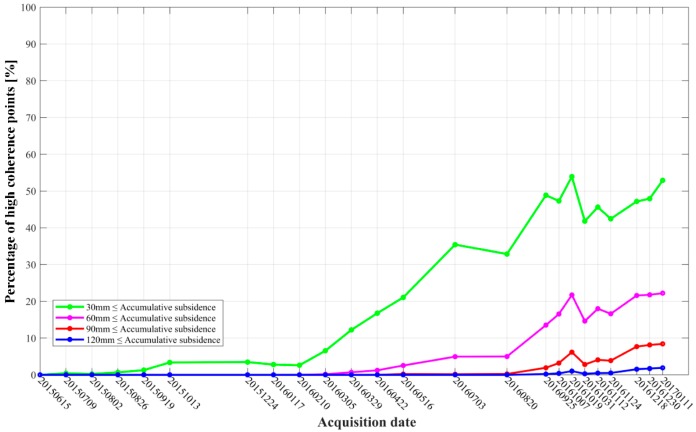
Percentage of accumulated number of coherence points with subsidence in the rock salt mine (with reference to 15 June 2015).

To further interpret the mechanics, we analyzed the accumulated number of coherence points in Figure 7. The statistical result is shown in Figure 10. It can be easily seen that the jumping happened at 10 February 2016, when the accumulated subsidence was above 30 mm, indicating a nearly 8-month period of stable surface condition. During the first 8 months, the subsidence was lower than 30 mm, which was mentioned as the time lag above. Since then, the number keeps increasing until 11 January 2017. From 5 March 2016, the increasing of the number of the high coherence points with subsidence above 60 mm began. Until January 2017, the accumulated number accounted for about 22%. From May 2016, the number of high coherence points, with subsidence above 90 mm and 120 mm, started increasing until January 2017, accounting for 8% and 2%, respectively. As mentioned above, the suggested reasons for the long time of the lagging phenomenon was mainly related to the rock salt dissolution delay and a much deeper mining depth in the process of drilling solution mining [3].

It can be seen from Figure 10 that the shape of the statistical curve performs to be a waveform curve. According to the principle of hydraulic transmission, in the process of drilling solution mining, the pressure generated by the new injected fresh water played a supporting role on the roof of the cavern; accordingly, the subsidence of the ground surface would be decreased [40]. This is also one of the reasons why the statistical curve is flat. With abundant precipitation in the rock salt mine area throughout 2016, the underground volume of the shallow aquifer was increased by the supplement of the nearby river network; thus, a small uplift of the ground surface appeared. This is another reason why the fluctuations occurred in the statistical curve. For example, from August to October 2016, high temperature and low rainfall dominated in the test area, as shown in Figure 9. Combined with the water evaporation in shallow aquifer, the ground surface subsidence was more serious. Accordingly, the cumulative number of pixels, with the subsidence greater than 30 mm, 60 mm, 90 mm, and 120 mm, increased rapidly, as shown in Figure 10. However, from 19 October 2016 to 31 October 2016, the external temperature decreased continuously. Combined with the large amount of precipitation, the slow dissolution rate of rock salt lead to the small uplift of the ground surface. Consequently, the cumulative number of ground surface subsiding pixels was significantly reduced for this period.

**Figure 11:**
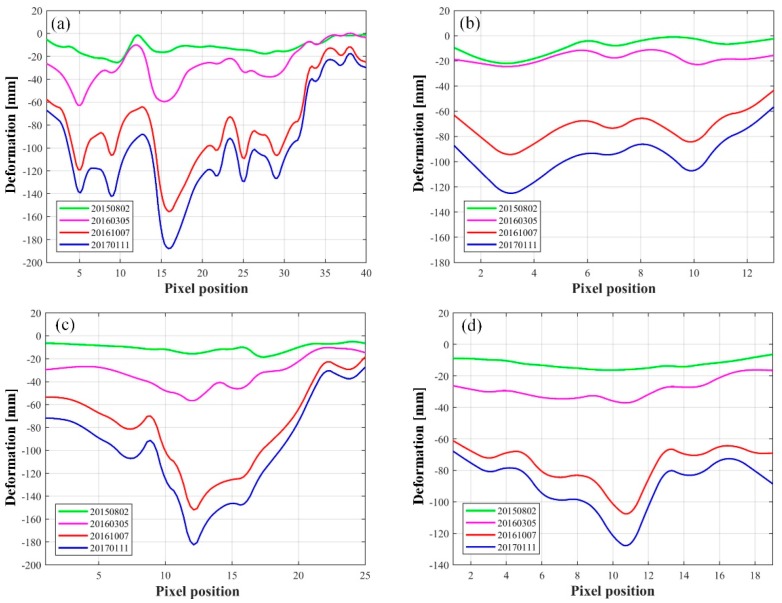
Profiles of the subsidence bowl in Figure 7. (**a**) Along the l1 direction, (**b**) along the l2 direction, (**c**) along the l3 direction, and (**d**) along the l4 direction.

To further discuss and analyze the growing process of the typical subsidence bowls detected in the test mine, the profile analysis along the transversal and longitudinal directions (see the transversal line *l*1 and longitudinal lines *l*2, *l*3 and *l*4 in Figure 7) was carried out. The results are shown in Figure 11. We can see that obvious multi-peak phenomenon occurred along the transversal and longitudinal directions. According to our measurements, the peak subsidence along the *l*1 direction was 140 mm, 142 mm, 191 mm, 129 mm, and 128 mm on the fifth, ninth, 16th, 25th, and 29th pixels, whereas 129 mm and 113 mm at the third and 10th pixels along *l*2 direction. The maximum subsidence of 191 mm and 137 mm were detected at the 12th and 11th pixels along the *l*3 and *l*4 directions, respectively. The multi-peak phenomenon along the transversal and longitudinal directions was mainly related to the drilling solution mining method based on connected well groups and its comprehensively multi-direction advancing mode. 

### 4.3. Accuracy Assessment

**Figure 12:**
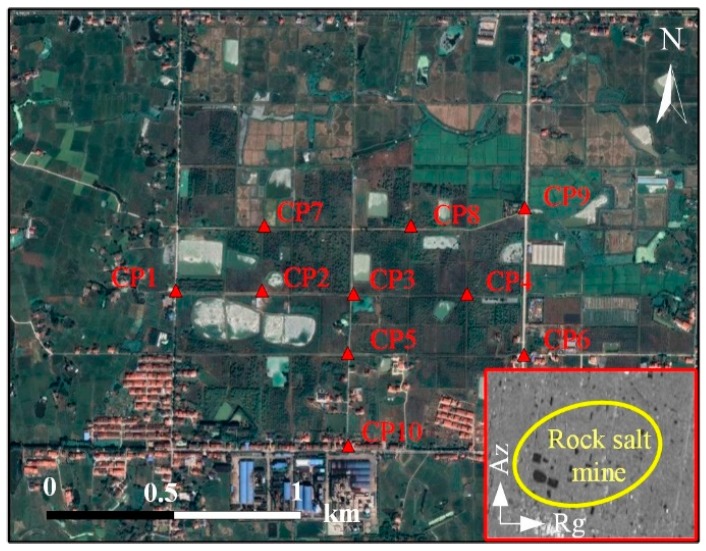
Locations of the benchmarks in the rock salt mine (the corresponding amplitude images are shown in the red rectangle in the southeast corner).

In order to verify the reliability of the monitoring results obtained by SBAS technology in this work, an in situ leveling method was carried out to compare with the obtained InSAR measurements. The locations of leveling points (CP1 to CP10) are marked with red solid rectangles in Figure 12. To perform an accurate comparison, we transferred the generated LOS deformation into vertical displacement according to Equation (3) and extracted the measurements that coincided temporally with our SAR acquisition dates. 

**Figure 13:**
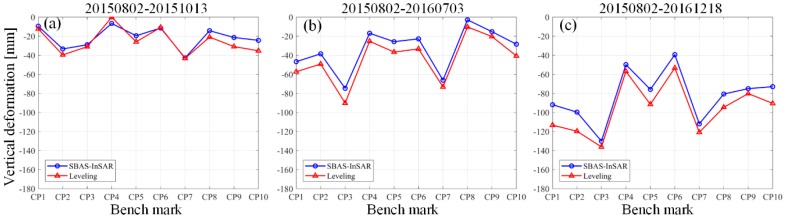
Times-series deformation results compared with leveling measurements on the benchmarks (the locations of CP1 to CP10 are shown in Figure 12). (**a**) from 2 August 2015 to 13 October 2015. (**b**) from 2 August 2015 to 3 July 2016. (**c**) from 2 August 2015 to 18 December 2016.

Figure 13 shows the comparison results. Obviously, the leveling points of the mining area are continuously subsiding during the period of observation. The most serious subsidence occurred at CP3 in the rock salt mine, with a magnitude of 136 mm, which showed good consistency with the obtained SBAS measurements. According to our calculation, the final root mean square error (RMSE) of the rock salt mine is ±11 mm, accounting for 8% of the corresponding maximum deformation value. The result indicates that the SBAS results maintain a good consistency with that of the leveling measurements. It’s also verified that SBAS-InSAR is feasible in the time-series deformation monitoring of rock salt mines.

## 5. Conclusions

In this study, the SBAS-InSAR technique with Sentinel−1A imagery was used to obtain the spatial–temporal characteristics of the ground subsidence caused by drilling solution mining activities. To reveal the triggering mechanisms of the spatial–temporal ground subsidence, a typical rock salt mine in Hunan Province, China was detected, and its SBAS-derived time-series subsidence maps were obtained. The maximum cumulative subsidence was detected up to 199 mm. 

The mechanical deformational characteristics of the rock salt mine were obtained through analyzing the time-series deformation maps, the temporal variations of selected feature points, the cumulative number of the coherence points, and the profiles of the subsidence bowls. Spatially, the distribution of the subsidence in the rock salt mine appeared as discrete strip-shaped in the northwest part and an overall sheet-like shaped distribution in the central and southeast part. Furthermore, the subsidence bowls were with multiple peaks along the transversal and longitudinal directions. This is related to the drilling solution mining method based on connected well groups, and its comprehensively multi-direction advancing mode. Temporally, the cumulative deformation variation curve of the rock salt mine showed a waveform characteristic, with a time lag of 8 months. The suggested reasons for this were that the pressure generated by the new injected fresh water played a supporting role on the roof of the cavern the large depth and thickness of the rock salt mine, and the process of rock salt dissolution induced the time delay in a combined manner. In addition, according to our measurements, the subsidence was greatly affected by the solvent temperature during the drilling solution mining process; thus, it showed obvious seasonal fluctuations. The reasons were supposed as the variations of the dissolution rate for mirabilite and thenardite. The high temperature in warm seasons accelerated the dissolution of rock salt, which led to the increase of subsidence. In contrast, the low temperature in cold seasons suppressed the process of water dissolution, inducing the slow or even uplift trend of the deformation.

Compared to the field leveling deformation measurements, the final accuracy was estimated to ±11 mm. The good consistency with the field measurements shows the feasibility and reliability of the SBAS technology and Sentinel−1 imagery in the application for the rock salt mine monitoring.

## Figures and Tables

**Table 1:** List of the interferometric pairs and their parameters (Ascending).

Drilling Water-Soluble Rock Salt Mine (Orbit No. 11)							
No.	Acquisition Date (yyyy/mm/dd)	Vertical Baseline (m)	Temporal Baseline (days)	No.	Acquisition Date (yyyy/mm/dd)	Vertical Baseline (m)	Temporal Baseline (days)
0	2015/06/15	26.89	−216	12	2016/05/16	−15.15	120
1	2015/07/09	88.17	−192	13	2016/07/03	−19.95	168
2	2015/08/02	1.87	−168	14	2016/08/20	22.27	216
3	2015/08/26	−36.04	−144	15	2016/09/25	−55.61	252
4	2015/09/19	−33.99	−120	16	2016/10/07	−21.78	264
5	2015/10/13	43.37	−96	17	2016/10/19	57.01	276
6	2015/12/24	121.57	−24	18	2016/10/31	54.31	288
**7**	**2016/01/17**	**0**	**0**	19	2016/11/12	42.52	300
8	2016/02/10	95.00	24	20	2016/11/24	0.96	312
9	2016/03/05	−23.73	48	21	2016/12/18	−20.47	336
10	2016/03/29	−48.43	72	22	2016/12/30	20.89	348
11	2016/04/22	39.75	96	23	2017/01/11	71.62	360

**Table 2:** Solubility of thenardite at different temperatures (g/100 g H_2_O).

Mineral	Temperatures (°C)										
	0	10	20	30	40	50	60	70	80	90	100
Thenardite (Na_2_SO_4_)	5.0	9.0	19.4	40.8	48.8	46.7	45.3	44.1	43.7	42.9	42.5
